# Strategies for targeted gene delivery using lipid nanoparticles and cell-derived nanovesicles

**DOI:** 10.1039/d3na00198a

**Published:** 2023-07-07

**Authors:** Dong-yup Lee, Sivashanmugam Amirthalingam, Changyub Lee, Arun Kumar Rajendran, Young-Hyun Ahn, Nathaniel S. Hwang

**Affiliations:** a School of Chemical and Biological Engineering, Institute of Chemical Processes, Seoul National University Seoul 08826 Republic of Korea nshwang@snu.ac.kr; b Interdisciplinary Program in Bioengineering, Seoul National University Seoul 08826 Republic of Korea; c Bio-MAX/N-Bio Institute, Institute of Bio-Engineering, Seoul National University Seoul 08826 Republic of Korea; d Institute of Engineering Research, Seoul National University Seoul 08826 Republic of Korea

## Abstract

Gene therapy is a promising approach for the treatment of many diseases. However, the effective delivery of the cargo without degradation *in vivo* is one of the major hurdles. With the advent of lipid nanoparticles (LNPs) and cell-derived nanovesicles (CDNs), gene delivery holds a very promising future. The targeting of these nanosystems is a prerequisite for effective transfection with minimal side-effects. In this review, we highlight the emerging strategies utilized for the effective targeting of LNPs and CDNs, and we summarize the preparation methodologies for LNPs and CDNs. We have also highlighted the non-ligand targeting of LNPs toward certain organs based on their composition. It is highly expected that continuing the developments in the targeting approaches of LNPs and CDNs for the delivery system will further promote them in clinical translation.

## Abstract 2

This review article discusses the latest formulation methodologies and strategies for the targeted delivery of lipid nanoparticles and cell-derived nanovesicles to achieve efficient gene transfection with minimized side effects.

## Graphical abstract

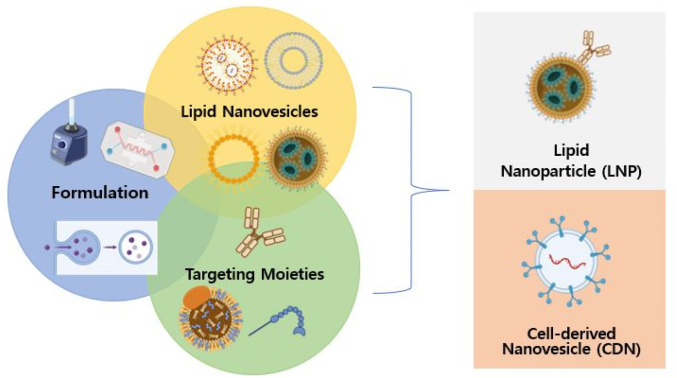

## 1. Introduction

Gene therapy has fascinated researchers since its inception, as the introduction of nucleic acid sequences or genome-editing sequences can alter the expression of the target gene and gene editing.^1–3^ However, many challenges remain unanswered for the delivery of genes *in vivo*, specifically, gene stability in the formulation and the complex milieu as it has to pass through mechanical, biological, and immunological obstacles without getting neutralized or degraded. Many concerns exist in the use of viral vectors for delivering a gene of interest, even though they would provide stability and possess higher transfection efficiency. Concerns include controlled overexpression of the target gene, difficulties in large-scale production, limited availability of target gene packaging (limited to 5 kb for adeno-associated viruses), carcinogenicity, and random insertion of the viral genome.^4–6^

During the last few decades, researchers have shown much interest in the use of non-viral delivery of target genes to the cells, particularly in the use of liposomes, lipid nanoparticles, polymeric micelles, and nanovesicles. These materials have a greater advantage over viral vectors in terms of higher packaging capacity, ease of modulating the structural properties, and large-scale production.^6^ With the advent of a lipid nanoparticle-based vaccine for COVID-19, it has proven that the lipid nanoparticles (LNPs) can be prepared on a large scale with proven safety.^7,8^ Another advantage of using LNPs is the use of ionizable cationic lipids that provide an escape from the endosomes and they release the cargo in the cytosolic area, thereby protecting the gene from neutralization/degradation in intracellular trafficking that ultimately leads to higher transfection efficiency.^9,10^ On the other hand, cell-derived nanovesicles (CDNs) are rapidly emerging as a carrier to overcome the shortcomings of LNPs.^11^ CDNs have the benefit of having lower immune recognition, and they could also be modulated at the donor cells providing a multitude of advantages for their use in gene delivery.^12^ However, it is important that transferring genes to specific areas requires targeting.^13,14^ Various targeting strategies for LNPs and CDNs have been explored in the last decade.

In this review, we summarize the current formulation methods for LNPs and CDNs, including the mechanism of gene encapsulation. Furthermore, we focused on the targeting strategies of LNPs and CDNs to the target site. The major focus of this review is to highlight the emerging strategies utilized for the targeting of lipid nanoparticles and CDNs. We hope that this review article would provide invaluable insights into the trending target approaches and instigate new ideas for efficiently delivering the cargo to the target site.

## 2. Formulation methods

### 2.1 Formulation methods of lipid nanovesicles

The ‘lipid nanovesicle’ is a broad term that includes lipid nanoparticles, liposomes, niosomes, transferosomes, ethosomes, and transethosomes.^15^ Vesicles are structures that have a core surrounded by one or more bilayer membranes, which are usually self-assembled in the presence of water. These vesicles can either be obtained endogenously from cells or synthesized using various methods. The bilayers of the synthesized vesicles are mostly made up of phospholipids, thereby closely resembling natural cell membranes.^16^ Thus, these lipid vesicles are usually termed liposomes, named after the Greek words *lipo* and *soma* meaning fat bodies. The liposome was described as early as 1962 by Alec D. Bangham and was suggested for use as a drug delivery system.^17–19^ These vesicles function as true compartments that could effectively hold the ionic concentration gradients and can act as organelles, thereby acting as efficient carriers for drugs and other molecules. With time, the basis of liposomes has served to design and fabricate various other lipid nanovesicles with various modifications and physicochemical properties. Based on the type of carrier systems, modifications, and functionality, lipid nanovesicles have been assigned various names such as niosomes, transferosomes, ethosomes, and transethosomes, apart from the actual liposomes.^16^ Various other nomenclatures have also been used to denote a multitude of liposomes.^20^ These modified liposomes exhibit better chemical stability, improved drug loading, reduced immunogenicity, and enhanced biocompatibility. Niosomes are lipid vesicles in which the phospholipids were substituted with surfactants such as Tween, Brij and Span.^21^ The transferosomes, which are deformable liposomes, had molecules to enhance penetration, were much smaller in size and also exhibited elasticity. All these properties greatly increased their drug delivery efficiency and they were also used to deliver peptides and genes.^22^ Ethosomes are lipid nanovesicles that are made up of up to 50% ethanol. The presence of ethanol has allowed the delivery of lipophilic and hydrophilic drugs, which was a hindrance when non-ethanol-based lipid nanovesicles were used. Ethosomes are mostly used for transdermal delivery application of various drugs.^23,24^

For synthesizing such varied types of lipid nanovesicles, various preparation techniques are being used. Each technique has its advantages and disadvantages. Certain techniques provide feasibility for large-scale production of lipid nanovesicles whereas certain other methods provide better homogeneity. This section will discuss briefly some of the synthesis methods that are being employed for lipid nanovesicle production, their disadvantages, and their advantages. The various synthesis methods can be grouped as follows:

(a) Conventional methods

(b) Microfluidic approach

(c) Compressed fluid-based methods

#### 2.1.1 Conventional methods

**Fig. 1:**
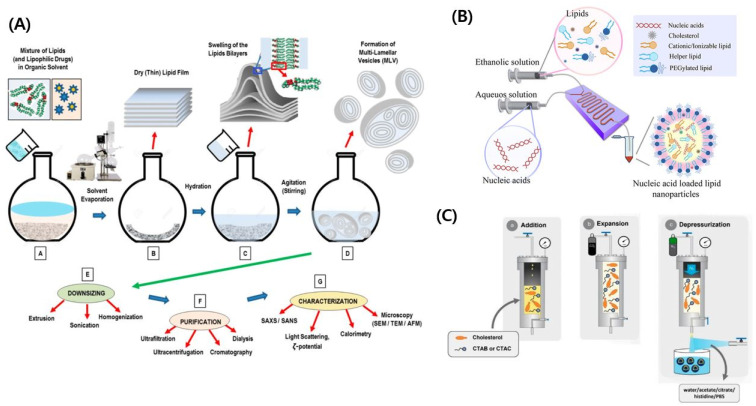
(A) Schematic illustration of the conventional nanovesicle formulation method. Reproduced with permission (Lombardo *et al.* 2020) from MDPI.^217^ (B) Schematic illustration of the microfluidic approach.^218^ Reproduced with permission (Ali *et al.* 2021) from American Chemical Society.^218^ (C) Schematic illustration of compressed fluid-based methods.^50^ Reproduced with permission (Ballell-Hosa *et al.* 2022) from MDPI.^50^

Conventional methods utilize the traditional approach of dispersing the organic phase containing the phospholipids into an aqueous phase at an optimized rate, subjecting this mixture to stirring or vortexing conditions, thus leading to the nanovesicular self-assemblies (Fig. 1A).^20,25,26^ However, these methods lead to the non-uniform size distribution of lipid nanovesicles. A modification to these methods is the heating of the reaction mixtures at around 40 °C with the option of adding ethanol, edge activators, permeation enhancing agent, and so on.^27^ This process is commonly called ethanol injection and can give rise to deformable lipid vesicles. The other commonly used conventional method is the Bangham method, which is technically a thin film hydration method. Herein, the phospholipids can be dissolved in an easily evaporable solvent such as chloroform, methanol, ethanol, and so forth, and the solvent is evaporated.^19^ This leaves a residue of thin film on the walls of the container. This thin film of phospholipid is exposed to water or other aqueous phases and allowed to swell under aqueous conditions. Thus, hydration process leads to the self-assembly of the phospholipids into lipid vesicles; however, the vesicles have a non-homogeneous size distribution. Thus, this thin film hydration method needs further processing such as sonication or membrane extrusions to obtain smaller and uniform vesicles. Even then, this process suffers from low drug loading; however, this can be improved by a repeated freeze-thawing process.^28,29^ Furthermore, the difficulty of reproducibility hinders this simple process in large-scale production. Certain techniques like reverse phase evaporation can enhance drug encapsulation into the lipid vesicles. The lipid phase is carried in organic solvents such as ethers, and a small volume of water is added to this organic phase. This leads to inverse micellar formation and when subjected to organic solvent removal, large unilamellar vesicles will be formed.^28,29^ One of the major concerns in this technique is the complete removal of solvent which is cumbersome. Similarly, a solution of detergent and lipid micelles can be fabricated, and the depletion of detergent will lead to the self-assembly of the phospholipid bilayer leading to vesicle formation.^30,31^ Again, this detergent depletion technique also leads to difficulties in the complete removal of the detergent in the final formulation. Most of these conventional methods lead to heterogeneous final yields that hinder consistent large-scale productions. Furthermore, almost all of these processes give larger vesicles, which need further sizing measures such as agitation by ultrasonication, mechanics, extrusions, or homogenization.^32^

#### 2.1.2 Microfluidic approach

To address the issues of heterogeneous particle distribution, precise drug loading, and complex vesicular assemblies, microfluidics can be utilized to form vesicle droplets (Fig. 1B).^33^ With current advancements in microfluidic chip fabrication methods, it is possible to load multiple drugs into the same vesicular assembly.^34^ By using various microfluidic techniques such as hydrodynamic flow focusing, staggered herringbone micromixers, multi-inlet mixers, impinging jet mixers, and so forth, researchers can have greater control over the flow and mixing rate of each component during the nanovesicle assembly and can obtain the vesicles as needed.^35^ Various drugs, genes, and proteins have been loaded into lipid vesicles using such microfluidic assemblies. With techniques like laser etching and photolithography, it is further possible to reduce the microfluidic chamber sizes.^36–40^ By tuning the flow and mixing rate of the lipid and aqueous solutions in a double spiral micromixer, researchers were able to change the rigidity of the nanovesicles.^41^ Furthermore, some of the microfluidic-based lipid nanovesicle assembly lines could handle around 1200 ml per hour, indicating a high throughput process.^42^ To further enhance the rigidity, a three-stage microfluidic assembly design was used to coat the nanovesicles with PLGA shells. With the cost of chip fabrication greatly reduced, it is economically viable to utilize multiple microfluidic nanovesicle assembler chips running simultaneously, to increase the yield in short timespans.^43,44^ Furthermore, with the ability to customize, design, and fabricate the chips in shorter times, a more patient customized approach can be made to tailor the lipid nanovesicles according to the needs. Microfluidic chips and analytical tools such as a laser spectrometer and laser diffraction particle size analyzer can be easily integrated to perform real-time quality control, thereby improving the final product.^45–47^ But, re-use of the chips for repeated nanovesicle assembly would be a concern because these chips would take more effort to clean for further use making them disposable assembly machines, as humanity moves towards sustainable solutions. However, considering the advantages over disadvantages, it might be plausible that microfluidics would dominate the large-scale production of lipid nanovesicles.

#### 2.1.3 Compressed fluid-based methods

Another novel and green solvent-based approach for synthesizing lipid nanovesicles is the compressed fluid-based technique. Herein, carbon dioxide (CO_2_) is mostly used in its compressed form due to its easy availability, being economically cheap, and recyclability.^48^ When CO_2_ is subjected to supercritical temperature and pressure, it can act both as a liquid and a gas. This property can be utilized to reduce the use of toxic solvents that are difficult to remove from the final formulations, and also a supercritical process can dramatically reduce the production times.^49^ Furthermore, the supercritical process can preserve the native structure and properties of the phospholipids, proteins, and drugs in a very efficient way, thereby preserving the potency of the load inside the lipid nanovesicles for maximum therapeutic outcome. Often, compressed fluid-based technology is used in combination with an already existing method such as the reverse phase evaporation or injection method. However, compressed fluid-based systems require higher pressurization and temperatures of around 60 °C for complete solubilization of lipids in the compressed fluid. To overcome this depressurization of an expanded liquid organic solution (DELOS-susp), a system was developed, wherein various membrane components are dissolved in the organic solvents.^50^ Then, CO_2_ is compressed into these phospholipid organic solvent mixtures and the solvent system is expanded. Then, depressurization is carried out over the aqueous phase which leads to the formation of uniform lipid nanovesicles (Fig. 1C).^51,52^ This process is advantageous compared to other compressed fluid-based methods, as it works under a reduced pressure and at a temperature of around 30 °C. Using this process, various drugs and peptides have been efficiently loaded into nanovesicles. This method also allows for an easier scale-up of the production process, thereby leading to mass production.

### 2.2 Formulation methods of gene delivering lipid nanovesicles (LNPs)

**Fig. 2:**
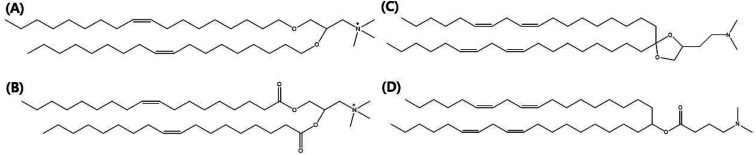
Molecular illustrations of cationic lipids. The permanently cationic lipids (A) DOTMA (1,2-di-*O*-octadecenyl-3-trimethylammonium propane) and (B) DOTAP (18:1 TAP) and the ionizable cationic lipids (C) Dlin-KC2-DMA and (D) Dlin-MC3-DMA are the most commonly used lipids for lipid nanoparticles.

In this review, we focus on lipid nanovesicles that deliver genes which are commonly called as lipid nanoparticles (LNPs). With the advent of COVID-19, LNPs have proven to be a promising gene delivery system. Genes are composed of nucleic acids and they are negatively charged.^53^ Therefore, cationic lipids are needed to deliver genes except for a few cases of formulation.^54^ Positively charged cationic lipids can be classified into two, which are ionizable cationic lipids and permanently cationic lipids. The difference between ionizable cationic lipids and permanently cationic lipids is the number of functional groups in the amine group, and the different effective charge due to pH change. Tertiary amines are cationic only at an acidic pH and quaternary amines are cationic at all pH values.^55^ As can be seen in Fig. 2(A) and (B), permanently cationic lipids have quaternary amines, and ionizable cationic lipids have tertiary amines as can be seen in Fig. 2(C) and (D). There is a slight structural difference between the two.^55,56^

**Fig. 3:**
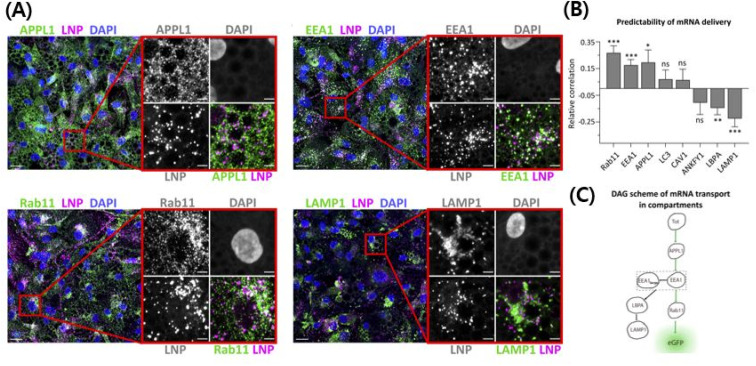
Use of ionizable cationic lipids provides an escape from the endosomes and they release the cargo in the cytosolic area, thereby protecting the gene from neutralization/degradation in the endosomal pathway.^215^ The intracellular trafficking demystifies the pathway that ultimately leads to higher transfection efficiency with less toxicity. (A) High-resolution confocal images showing cy5 mRNA encapsulating LNPs in the endosomal pathway. (B) The correlation between efficiently transfected LNPs and the endocytosis pathway. Rab11, EEA1, and APPL1 have a positive correlation with transfection efficiency of LNPs, while LBPA and LAMP1 have a negative correlation. Other endosomes do not have a correlation. (C) The scheme of the LNP's endocytosis pathway to explain the fact that efficient LNPs go to specific endosomes. Reproduced from (Paramasivam *et al.* 2021) with permission from *J. Cell Biol.*^215^

Most gene-delivering LNPs employ ionizable cationic lipids to encapsulate nucleic acids.^57^ A few gene-delivering LNPs utilize permanently cationic lipids, and even some LNPs do not have cationic lipids in lipid compositions.^57^ As can be seen in Fig. 2, LNPs using ionizable cationic lipids go to early or recycling endosomes, while permanently cationic lipids go to late endosomes or lysosomes (Fig. 3).^215^ Therefore, the formulation process of LNPs is also varied, although they share common characteristics such as delivering genes. Before identifying the targeting strategies of LNPs, we need to figure out what methods are used in the process of making LNPs.

#### 2.2.1 Self-assembly

Self-assembly is a term that can usually refer to pipetting, vortexing, and ethanol dilution. When using cationic lipids to formulate lipid nanoparticles, pipetting, vortexing, and ethanol dilution all have a similar mechanism. Hydrophilic drugs, nucleic acids which are genes, are loaded within the inner hydrophilic cavity by dissolving them in a buffer.^105^ Lipids are dissolved in the organic solvent while nucleic acids are dissolved in an aqueous buffer.^105^ Diffusion of alcohol and water across the interface induces the lipid to precipitate and formulate into lipid nanoparticles.^113^ Pipetting and vortexing are designed to formulate small and medium-sized particles, respectively, while microfluidic chips are capable of formulating smaller lipid nanoparticles and producing large-scale lipid nanoparticles.^114^

The difference in the viscosity of solvents among self-assembly methods is a unique formulation process worth paying attention to. Chen *et al.* first dissolved additional lipids in tetrahydrofuran and then mixed them with other lipid components in ethanol, followed by rapidly mixing with aqueous mRNA solutions.^95^ Tetrahydrofuran has a viscosity of 0.48 cP at 25 °C and ethanol has a viscosity of 1.1 cP at 25 °C.^115,116^ Viscosity is a measure of a fluid's resistance to flow, which means the higher the viscosity, the slower the solvent flows. Solutes in different solvents, therefore, flow in different speed. Chen *et al.* utilized the principle and developed a selective organ targeting LNP platform.^116^

#### 2.2.2 Thin film hydration

Thin film hydration is a traditional technique to formulate lipid nanoparticles.^117^ But it is not common for gene-delivering LNPs. Lipids mixed in volatile solvents are dried in a flask as a thin film.^118^ Then, the thin film undergoes the hydration process with an aqueous buffer by sonication. The obtained thin films are formulated into nanoparticles because lipids are amphiphilic, but the size distribution is too wide. Therefore, to produce desirable and stable LNPs, size-controlling methods such as extrusion, sonication, and high-pressure homogenization are needed.^119^ To conjugate targeting moieties, decreasing the multilayer assembly and reorganizing of targeting ligands and lipids is required. Freezing and thawing is one option for this.^117–120^

#### 2.2.3 Microfluidics

Thin film hydration is less preferred than microfluidic methods and self-assembly methods. Lipid thin film hydration has a lower yield of gene-delivering lipid nanoparticles due to the fragility of the nucleic acids. Furthermore, microfluidics obtains greater yield with the improvement of technology.^121^

A microfluidic chip is the representative technique for formulating gene-encapsulated lipid nanoparticles. COVID-19 (Coronavirus disease of 2019) mRNA vaccines were made of lipid nanoparticles formulated by microfluidics.^104^ Among the gene-delivering LNPs made in the past five years, those with ionizable cationic lipids use microfluidic chips for their formulation. For the vaccine industry, LNPs could be prepared on a large scale by microfluidics.^105^ Another important factor in the vaccine industry is that using microfluidic chips can make LNPs smaller. It allows LNPs to target lymph nodes efficiently, allowing vaccines to effectively activate immune cells without severe side effects.^78^ Which property of a microfluidics chip makes LNPs smaller? The sooner the LNP is diluted from ethanol, the smaller it is. To achieve supersaturation of lipid monomers, it is suggested that the lipid–ethanol solution and the aqueous buffers are mixed in a rapid phase, resulting in the homogeneous nucleation of nanoparticles.^106^ The precipitation occurs faster than particle formation, and results in the formation of small-sized particles.^106^

**Fig. 4:**
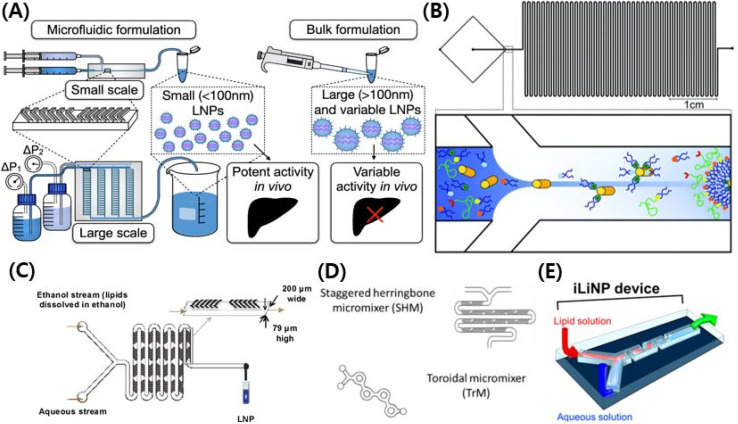
(A) Schematic illustration of microfluidic chips formulating lipid nanoparticles. Reproduced with permission (Shepherd *et al.* 2021) from Shepherd *et al.* American Chemical Society.^216^ (B) Schematic illustration of T-junction mixers with hydrodynamic flow focusing (HFF).^108^ Reproduced from with permission (Krzysztoń *et al.* 2017) from the Royal Society of Chemistry.^108^ (C) Schematic illustration of chaotic micromixers.^109^ Reproduced with permission (Zhigaltsev *et al.* 2012) from American Chemical Society.^109^ (D) Schematic illustration of bifurcating mixers.^105^ Reproduced with permission (Roces *et al.* 2020) from Pharmecuetics.^105^ (E) Schematic illustration of baffle mixers.^110^ Reproduced with permission (Kimura *et al.* 2018) from American Chemical Society.^110^

There are many kinds of LNP manufacturing microfluidic chips.^35^ The typical types of microfluidic chips are T-junction mixers, hydrodynamic flow focusing (HFF), chaotic micromixers, bifurcating mixers, and baffle mixers.^107–109^ A prototype of microfluidics chips is the T-junction mixers, which are T-shaped, and they were used to formulate enough LNPs for primate experiments.^107^ T-Junction mixers allow bulky, rapid mixing but they are poor at controlling the particle size.^107^ An HFF is a T-junction mixer that has a flat cross-shaped pattern.^108^ Two aqueous solutions from perpendicular tubes collide with each other and become mixed with an organic solution that flows in the center.^108^ LNPs from HFF have higher encapsulation efficiency than those from batch mixing, but it cannot control the size of the LNPs.^108^ A chaotic mixer uses a herringbone structure to create chaotic advection of the laminar flow.^109^ Chaotic mixing control LNPs in size by changing the flow rate (FR) and flow rate ratio (FRR).^109^ Concerning chaotic mixing design, increasing the FR and FRR achieve production of small-sized LNPs.^109^ The baffle mixer has a simple two-dimensional structure to compensate for the shortcomings of the chaotic mixer that easily clogs LNPs.^110^ To mix a solution rapidly, baffle mixers have turns in a row which help baffle mixers control the LNPs well in size.^110^ However, the chaotic mixer and the baffle mixer are both inappropriate for large-production.^110^ Bifurcating mixing renders a high output rate of the chips, and it leads to large-scale production.^105^ A bifurcating mixer has a toroidal-shaped structure, and the shape helps two other solutions to collide with each other.^105^ Through this process, encapsulation efficiency and control in size were kept in good shape with enhanced production rates (Fig. 4).^105^

#### 2.2.4 Extrusion

Extrusion is a process of introducing substances into an extruder so that they can be mixed more homogeneously.^111^ Extrusion is one of the strong methods to formulate cell nanovesicles that can replace natural extracellular vesicles.^111^ Such a method can also be applied to LNPs.^79^ Nucleic acids were mixed with lipids diluted in ethanol, and extruded through an extruder; additional lipids were inserted, followed by a buffer change to PBS.^79^ Extrusion helps the nanoparticles spread evenly and the size is matched to the extruder's pore size.^112^

## 3. Targeting strategy of lipid nanoparticles

In the case of gene delivery, decreasing the potential for off-target delivery has been an ongoing major issue. Therefore, in designing gene-delivering nanocarriers it is essential to develop a viable strategy for the carriers to access the target site. The first attempts at cell-specific nanocarrier targeting occurred in 1980, by conjugating an antibody or protein A to a liposome.^122^ Since then, various types of targeting moieties such as transferrin for blood–brain barrier passage, glucose for tumor targeting, and integrin for inflammatory bowel disease (IBD) to name a few, have been brought up.^123–125^ Of the many types of active targeting strategies proposed so far, we can largely classify the methods of targeting into two classes: ligand-mediated targeting and targeting *via* the physicochemical characteristics of the nanoparticle. The following section will discuss the different methods of targeting in detail.

### 3.1 Ligand-mediated targeting

Ligand-mediated targeting refers to targeting a specific surface receptor of a cell directly and/or indirectly. Concerning LNPs, the methods of incorporating targeting ligands into the lipid surface vary from the traditional post-insertion method to newer methods like anchored secondary scFv enabling targeting, or ASSET henceforth.^61^ The following section will cover some of the strategies developed to integrate ligands into LNP carriers.

#### 3.1.1 Post-insertion of targeting ligands into preformed lipid nanoparticles

The post-insertion strategy of ligand incorporation (Table 1) is one of the more classical strategies, with the first instance of post-insertion incorporated mouse monoclonal antibody for human β_2_-microglobulin and protein A from *Staphylococcus* into preformed liposomes by reacting phosphatidylethanolamine with *N*-hydroxy succinimidyl-3-(2-pyridyldithio)propionate to form a dithiol structure and replacing the pyridine with an antibody *via* thiol chemistry.^122^ Recently, antibodies are being utilized not only for liposomes but for LNPs as well. For example, in a study concerning T-cell transfection, LNPs consisting of MC3 ionizable lipid, DSPC, cholesterol and PEG were conjugated with either human or murine anti-CD3 antibodies by the thiol–maleimide mechanism.^58^ Compared to untargeted LNPs, aCD3-LNPs were able to transfect over 80% Jurkat cells *in vitro* and 2–7% splenic and 2–4% circulating T cells *in vitro*. However, a point to be made was that the anti-CD3 antibody was shown to deplete T cells, and aCD3-LNPs induced the secretion of many types of cytokines. Another study also exploited the thiol–maleimide conjugation method by connecting an anti-CD4 monoclonal antibody with a PEG-maleimide linker.^84^ By converting the primary amine of the antibody to a sulfhydryl group using SATA and hydroxylamine, the new sulfhydryl group was able to connect to the maleimide group. This study also showed nearly 80% transfection efficiency of CD4^+^ T cells *in vitro*, and 60%, and 40% transfection of CD4^+^ T cells in the spleen and lymph node, respectively. Similarly, by conjugating anti-CD5 antibodies, Rurik *et al.* was able to target both CD8^+^ and CD4^+^ T cells and transfect them into fibroblast activation protein (FAP) specific CAR-T cells.^85^ The *in vivo* generated CAR-T cells were able to target FAP-expressing cells to repress fibrosis in cardiac injury models.

**Table 1:** Post-insertion of targeting ligands into LNPs

Receptors	Targeting ligands	Targeting strategy	Diseases	Reference
α4β7 integrin (inflammatory gut-homing leukocytes)	MAdCAM-1-Fc protein	DSPE-PEG-maleimide (one-pot)	Piroxicam-accelerated colitis (PAC)	80
			Inflammatory gut-homing leukocyte targeting	
Cd3	aCD3 mAb (*in vitro*)	DSPE-PEG-maleimide (one-pot)	CD3 phenotype T cell targeting in a tumor environment	58
	aCD3 F(ab′)2 (*in vivo*)			
Caveolae	Plasmalemma vesicle-associated protein (PV1)	DSPE-PEG maleimide (one-pot)	X, lung targeting	81
Lung endothelial cells	Anti PECAM-1 mAb	DSPE-PEG-Mal (mal post insertion)	Lung targeting	82
DEC205+ murine dendritic cells	DEC205 scFv	DSPE-PEG-Mal (one-pot)	X, spleen isolation after 24h of retro orbital injection	83
Cd4	aCD4 mAb	DSPE-PEG-Mal (mal post insertion)	CD4 phenotype T cell targeting	84
Splenic T cells	aCD5	DSPE-PEG-Mal (mal post insertion)	Cardiac fibrosis	85
Antigen specific CD8^+^T cells	pMHC1	DSPE- PEG2000-mal and conjugation with TCEP	Pr8 influenza virus	59
Osteoblast	CH6 Aptamer	Ch6-S-S-(SH_2_)_2_-OH → CH6-SH with DTT, DSPE-PEG-Mal → DSPE-PEG-CH6 (post insertion mal)	Metabolic skeletal disorders associated with impaired bone formation (*e.g.* osteoporosis)	86
Cerebrovascular of inflamed brain	VCAM-1 (CD106)	DSPE-PEG_2000_ azide	Glioblastoma (pathological permeability of the BBB)	96
Neutrophils	aCD177	Peptide H or peptide M-PEG_2000_-DSPE, m-PEG_2000_-DSPE	X	102
Ly6C + inflammatory leukocytes	Anti-Ly6C antibodies by ASSET (anchored secondary scFV enabling targeting)	DSPE-PEG-Ome (one-pot)	DSS colitis	60
Cd34 mAB for ASSET evaluation, Ly6C for DSS colitis	Anti-Ly6C antibodies by ASSET (anchored secondary scFV enabling targeting)	DSPE-PEG-carboxyl (carboxyl post insertion with EDC and NHS)	DSS colitis	61
Ly6C + inflammatory leukocytes	Anti-Ly6C antibodies by ASSET (anchored secondary scFV enabling targeting)	DSPE-PEG and ASSET	DSS colitis	62
EGFR	aEGFR (ASSET)	DSPE-PEG and ASSET	Tumor	63

Along with antibodies, modified proteins have been used as targeting ligands. Dammes *et al.*, took notice of the high-affinity conformation of integrin α_4_β_7_.^80^ Targeting integrin α_4_β_7_ itself has some limitations because it is expressed on various T cells in random populations. However, by changing the conformation, α_4_β_7_ can increase its affinity to a specific type of ligand. By designing a plasmid that connects mucosal vascular addressin cell adhesion molecule-1 (MAdCAM-1) extracellular D1 and D2 domains to an Fc region of rat IgG which was then conjugated to an LNP PEG chain using thiol–maleimide chemistry, the authors were able to successfully target the gut environment with high specificity to alleviate inflammatory bowel disease (IBD).

The recently acclaimed, “click chemistry” has also been used as a conjugation method. The first instances of click chemistry used copper as a mediator to conjugate azide and alkyne functional groups in a relatively simple condition. However, due to the toxicity of copper in the body, new methods of click chemistry have been developed. The strain-promoted alkyne–azide cycloaddition, otherwise known as SPAAC conjugates an unstable cyclooctyne group to an azide. The instability of the cyclooctyne induces the click reaction. In a recent study concerning click chemistry, the authors conjugated DSPE-PEG-DBCO with either sulfo-cy3-azide or (2-[4-(3-(*o*-tolyl) ureido)-phenyl]-acetyl)-l-leucyl-l-aspartyl-l-valinyl-l-propargylglycine-azide (LDV-azide). Interestingly, when the azide ligand was conjugated to a post-formulated LNP, the structure of the LNP broke into micelles, while when an already conjugated DSPE-PEG_(2000)_-triazole-ligand was post-inserted into the LNP mix, the particles showed a conserved spherical shape and an increase of <50% in the hydrodynamic diameter.^126^ This method of conjugating targeting ligands has been used in the treatment of acute brain inflammation.^96^ DBCO-PEG-NHS was incubated with antibodies against VCAM-1 and then conjugated to mRNA-encasing cationic LNPs with an azide moiety at the end of the PEG tails to make a VCAM-1 targeted LNP. When anti-VCAM antibodies were used as a targeting ligand, the uptake of the LNPs exceeded that when targeted by anti-TfR-1 and anti-ICAM-1 antibodies. It was shown that the VCAM-1 targeting antibodies were more prone to be taken up by the blood–brain barrier (BBB) endothelial cells. Thus, the authors proposed the potential of VCAM targeting in inflamed brains.

Recently a new one-for-all type of antibody conjugating method has been developed as well. Named anchored secondary scFv enabling targeting, or ASSET, this method applies protein modification as well antibody association to conjugate targeting antibodies to LNPs.^61^ The bacterial gene *nlpA* encodes lipoprotein-28 which can be used to anchor the ASSET system to the nanoparticle membrane. In short, ASSET consists of a signaling N-terminal, an *nlpA*-mediated lipoprotein, and an outward-facing scFv region.^127^ This scFv region can latch on to the Fc region of rat IgG2a antibodies, and thus any antibody that consists of a rat IgG2a Fc region can be used as a targeting ligand. This platform is advantageous due to the fact that it can be used to easily switch up the targeting antibody used, without chemical conjugation. The ASSET system was applied to deliver murine models of inflammatory bowel disease siRNA and mRNA and also was used to deliver CRISPR-Cas9 to treat glioblastoma and OV8 ovarian adenocarcinoma.^60,62,63^ Thus, the versatility of the ASSET platform could be exploited for various targeting LNPs.

#### 3.1.2 Direct conjugation of ligands to lipids

Tissue targeting LNPs can also be obtained by conjugating the targeting ligand directly to the lipids and it would utilized in LNPs formulation, thereby controlling the ligand density (Table 2). While connecting a targeting moiety to the end of the PEG tails of LNPs acts as a sort of biomimetic cell surface ligand and is a popular strategy, directly conjugating a ligand to lipids can also be used to target specific sites.^64,97^ For example, by exploiting the Ca^2+^ ion chelating ability of bisphosphonate (BP), Xue *et al.* were able to deliver BMP-2 mRNA to hydroxyapatite in the bone.^64^ Alendronate, a type of BP, was modified with *N*-acryloxysuccinimide to make alendronate acrylamide and then Michael addition produces alendronate amine cores. These cores were then conjugated with epoxy-terminated alkyl chains to make 21 types of BP lipids. The lipids were screened to find the most efficient targeting structure, composed of LNPs consisting of DOPE, cholesterol, and C14 chain PEG_(2000)_, and sent to the bone. When BMP-2 mRNA was incorporated into the LNPs and sent to the bone *via* intravenous injection, both the surface and the marrow showed elevated responses to BMP-2. Compared to normal LNPs and the PBS control, BP-lipid LNPs showed higher BMP-2 levels in both the bone marrow and at the surface of the bone.

**Table 2:** Direct conjugation of ligands to lipids

Receptors	Targeting ligands	Targeting strategy	Diseases	Reference
Monocytes in bone marrow	Bisphosphonate	Alendronate-bearing ionizable lipid (one-pot assembly)	X, bone targeting	64
Bone marrow mesenchymal stem cells	Bone homing peptide	Ionizable lipid having a head group of BHODA (or HODA or ODA) (one-pot assembly)	Osteoporosis	97
Langerhans cells, dermal DCs, and resident DCs in the skin-draining lymph node	Mannose (intradermal)	Man-cholesterolamine	Influenza virus	65
LSEC	Mannose	DSPE-PEG-mannose and then ApoE mixing	X, liver targeting	66
CD209/DC-SIGN and CD207/Langerin (human and mouse DCs and other hematopoietic cell populations)	Mannose	TriMN-Lip, MN-Lip, 1-*O*-carboxyl-2-*O*-phytanyl-3-*O*-hexadecane-*sn*-glycerol, the *O*,*O*-dioleyl-*N*-[3*N*-(*N*-methylimidazolium iodide)propylene] phosphoramidate, *O*,*O*-dioleyl-*N*-histamine phosphoramidate, and PEG-HpK	Tumor	98
Invariant natural killer T cells	α-Galactosylceramide	α-Galactosylceramide (replacement)	Tumor (melanoma)	99
Dendritic cells in a lymph node after SC	Mannose	PEG-mannose	Tumor (melanoma)	100
Dendritic cells	Mannose analogues	Mannose analogues	Tumor (melanoma)	101
Dendritic cell	Mannose	PEG-HpK and TriManlipo100	Tumor (melanoma)	103

Using self-amplifying RNA, Goswami *et al.* formulated an LNP with mannose-conjugated cholesterol.^65^ Starting with mannose succinate, 1-ethyl-3-(3-(dimethylamino)propyl)carbodiimide (EDC), and *p*-nitrophenol were added to form a mannose monoester. The newly formed monoester was then reacted with (3β)-cholest-5-en-3-amine to finally form mannose-connected cholesterol *via* the aminolysis mechanism. Mannose receptors such as C-type lectin receptors and Langerin receptors are highly expressed on antigen-presenting cells, and this in turn can be exploited as a target. When the anti-hemagglutinin vaccine was applied by dermal injection, the targeted LNPs were able to induce Th1 immune responses and activate CD8^+^ T cells *in vivo*. Another study applies this mannose conjugating strategy by formulating trimannosyl diether lipid (triMN-Lip) and mixing it with O, *O*-dioleyl-*N*-[3*N*-(*N*-methylimidazolium iodide)propylene] phosphoramidate and *O*,*O*-dioleyl-*N*-histamine phosphoramidate to form mRNA tumor vaccines (triMN-LPR).^98^ The triple mannose antenna was shown to associate better than single mannose LNPs in mouse DC2.4 cells, with an association of nearly 100% and 70%, respectively. Also, the triMN-LPRs showed higher immunization efficiency than the single-mannose LPRs. Similarly, mannose conjugated lipids were utilized for targeting melanoma.^100,101,103^

Dual ligand targeting including mannose could also increase the targeting properties of nanoparticles. In a study concerning selective LNP delivery to hepatocytes and liver sinusoidal endothelial cells (LSECs), the authors used apolipoprotein E (ApoE) incubation against LNPs to target low-density lipoprotein receptors in hepatocytes and LSECs.^66^ However, while ApoE incubation showed high targeting efficiency to hepatocytes, LSEC delivery was not as pronounced. To increase LSEC delivery efficiency, the authors lowered ApoE absorption by increasing the PEG levels of the nanoparticle. This, however, decreased the size of the LNPs which consequently induced elevated hepatocyte uptake due to a possible decrease in LSEC fenestrae association. This was solved by additionally adding mannose-PEG lipid to the LNP formulation. This difference in formulation changed the uptake percentage to 15% hepatocytes, 70% LSECs, and 15% Kupffer cells.

Conjugated lipids can be used as a targeting moiety and also as an adjuvant as well. α-Galactosylceramide (α-GC) is an immune-activating glycolipid that indirectly induces immune activation. The authors formulated α-GC, combined it with DOTAP, cholesterol, and mRNA and intravenously delivered the formulated particle.^99,128^ The formulated nanocarrier was taken up by antigen-presenting cells, presented to invariant natural killer cells (iNKs), and the iNKs in turn activated dendritic cells to initiate the immune response. When modified ovalbumin mRNA was delivered as a payload to mice affected with E.G 7-ovalbumin lymphoma and B16F10-ovalbumin melanoma, the ovalbumin mRNA expression caused the adaptive immune system to attack the tumor sites. When administered to E.G 7-ovalbumin bearing mice, modified mRNA α-GC LNPs caused 3 out of 7 mice to show complete tumor regression, while in B16F10-ovalbumin bearing mice the mice were able to survive 5.5 more days than the control mice.

### 3.2 Targeting without ligands

Active targeting has been an essential condition for targeted therapy for decades, but there is a big limitation in active targeting due to coated protein coronas.^129^ Active targeting leads to unexpected results in size, plasma concentration, pharmacokinetics, and intracellular pathway. Therefore, there are alternatives to active targeting. Endogenous lipid trafficking can be an alternative to active targeting.^68^

Lipid nanoparticles delivering genes are usually composed of cationic lipids, cholesterol, helper lipids, and PEG lipids.^130^ Several research groups conducted a study to target by changing one of the four components. The following section will discuss how lipid nanoparticles delivering genes can target specific organs without ligands.

#### 3.2.1 Cationic lipid modification

The role of cationic lipids is binding genes and facilitating endosomal escape.^130^ Due to various endocytosis pathways of different cells, endosomal escape of genes is affected by molecular properties of cationic lipids in cell targeting.^130^ Also, the modified cationic lipids can alter LNP tropism and can promote cell targeting. Since ordinary mRNA LNPs are taken up by the liver, LNPs targeting other organs such as the spleen, lymph node, or lung are of value (Table 3). When cationic lipids are synthesized, it is not clear why organs are targeted by modification. Therefore, it is reasonable to explain the possible correlation or speculative reason of each article.

**Table 3:** Cationic lipid modification

Target organ/cells	Chemical synthesis strategy	Targeting strategy	Diseases	Reference
B lymphocytes in the spleen	EDC coupling	OF-Deg-Lin	X	67
T lymphocytes in the spleen	EDC coupling	11-A-M (constrained)	X	68
Albumin associated micropinocytosis and endocytosis	EDCI	Alkyne (unsaturated) and ester groups into the lipid tails of Dlin-MC3-DMA	X	89
Macrophages containing antimicrobial peptides linked to cathepsin B in the lysosomes (MACs)	NaBH(OAc)_3_	Vitamin C	Sepsis	70
Spleen	NABH_4_	tB-UC18 [3,5-bis-(1-octadec-9-enylamino-methyl)-benzyl]-octadec-9-enyl-amine	X	87
		Unsaturation		
Liver	Sequential	Unsaturated thiol conjugated lipids promote endosomal escape	X	88
	Synthesis			
STING pathway immune cells	Amine/isocyanide/ketone of 1 : 1 : 1	Unsaturated lipid tail, a dihydroimidazole linker and cyclic amine head groups containing lipids (activation of the STING pathway)	Tumor (melanoma and HPV E7)	69
Lung	Michael addition reaction (amine and acrylate)	Lipids containing an amide bond in the tail	Tuberous sclerosis complex	71
Fetal liver	Michael addition (the polyamine cores and lipid epoxide)	Polyamines with epoxide terminated alkyl tails	X	72
APCs in lymph nodes	Michael addition (amine-bearing head and an acryloyl group containing aliphatic chain)	Combination of lipids	Melanoma	90

Lipid nanoparticles delivering genes target the liver for most of the processes. Dlin-MC3-DMA is a popular ionizable cationic lipid used for delivering genes, and therefore, there is a synthesis strategy modifying Dlin-MC3-DMA rather than synthesizing a new lipid. Miao *et al.* researched alkyne lipids that significantly increased membrane fusion to enhance mRNA release.^89^ The article showed that Dlin-Mc3-DMA having alkyne lipid tails, with protein corona mixing, has better protein expression in the liver by albumin-associated micropinocytosis.^89^ Lee *et al.* chemically synthesized 4A3-Cit as an ionizable cationic lipid. Synthesis by using unsaturated thiols showed improved transfection efficiency by 18-fold *in vitro*, and also a huge amount targeting the liver.^88^ Unsaturation, like by Miao *et al.* helps target the liver more efficiently. Vitelline vein injection of the LNPs by Riley *et al.* targets fetal liver.^72^ The targeted organ is decided by the structure of the polyamine-lipid core and the length of lipid epoxide, which are important for gene liver delivery.^72^

LNPs targeting other organs such as the spleen or lung are of value. Fenton *et al.* chemically synthesized new cationic lipids using EDC coupling and they succeeded in formulating lipid nanoparticles.^67^ OF-Deg-Lin is the cationic lipid Fenton *et al.* synthesized. Because OF-Deg-Lin has improved electrophilicity and ester linkages in OF-Deg-Lin generate a nontoxic fatty acid, it is targeted to B lymphocytes of spleens.^67^ Of-Deg-Lin mRNA LNPs are also delivered to the liver but protein translation does not proceed in liver cells.^67^ This implies that mRNA with OF-Deg-Lin survives the enzymatic conditions in the spleen, but not in the liver.^67^ The spleen is mainly composed of B cells and T cells.^131^ Targeting T cells in the spleen is as valuable as targeting B cells. Lokugamage *et al.* synthesized a constrained adamantane cationic lipid, forming an armchair structure, which is able to deliver siRNA to T cells *in vivo*.^68^ It is successfully targeted to T cells *via* natural trafficking, meaning an alternative to active targeting.^68^ Li *et al.* developed a new lipid nanoparticle platform for COVID-19. TB-UC18 which targets the spleen showed that saturation and length of the lipid tail affect transfection efficiency, meaning that the more unsaturated and longer the lipid tail is, the higher the expression in the spleen. On the other hand, lipid nanoparticles could deliver genes to the lung efficiently. Qiu *et al.* delivered tuberous sclerosis complex 2 (TSC2) mRNA in a model of lymphangioleiomyomatosis.^71^ N-series LNPs containing an amide bond in the tail selectively target mRNA to the mouse lung.^71^ The reason is the different compositions of protein coronas, and their tropism to the lungs.^71^

Targeting strategies are not limited to main organ targeting. Miao *et al.* developed lipid nanoparticle delivery of mRNA vaccines. In the article, antigen-presenting cell's maturation is induced by an unsaturated lipid tail and cyclic amine head groups *via* the intracellular stimulator of interferon gene (STING) pathway, and it shows antitumor efficacy.^69^ Hou *et al.* conjugated vitamin C to cationic lipid, allowing the specific accumulation of antimicrobial peptides and cathepsin B in macrophage lysosomes, which has a critical role in bactericidal activities.^70^ Chen *et al.* developed an LNP platform targeting lymph nodes, which can decrease side effects by efficiently targeting APCs.^90^ The platform showed efficacy in a melanoma model.^90^

#### 3.2.2 Cholesterol modification

Cholesterol plays a role in pulling the lipids to a liquid-ordered state.^130^ When combined with phospholipids with low melting temperatures, cholesterol helps decrease membrane fluidity and increase bilayer thickness.^130^ However, it works in the complete opposite way to high melting temperature phospholipids.^132^ Paunovska *et al.* conducted research by modifying cholesterol to target lipid nanoparticles.^73,74^

Paunovska *et al.* formulated LNPs with esterified cholesterol which was much more efficient in gene delivery than regular cholesterol or oxidized cholesterol.^74^ CRISPR sgRNAs were efficiently delivered by LNPs containing cholesteryl oleate to liver endothelial cells *in vivo*.^73^

Another related research study about cholesterol modification was published in *Advanced Materials*.^74^ The LNP in the article targets liver microenvironmental cells five times more than hepatocytes.^74^ The position of the oxidation modification in cholesterol significantly impacts targeting efficiency. Sterol ring D-modified cholesterols modified target liver microenvironmental cells more than sterol ring B-modified cholesterols.^74^ These data suggest that modified cholesterols make LNPs deliver gene-editing mRNA to the liver microenvironmental cells efficiently.^74^

#### 3.2.3 Phospholipid modification or replacement

Phospholipids support stability during storage and circulation, which serves to support the structure.^130^ Phosphatidylserine promotes the endocytic activity of phagocytes and cellular internalization of enveloped viruses, and it is a well-known signaling molecule.^75^ Luozhong *et al.* formulated Dlin-Mc3-DMA-based LNPs containing phosphatidylserine and concluded that they help target secondary lymphoid organs.^75^ Gan *et al.* formulated LNPs containing constrained phospholipids by using adamantyl groups. They target liver endothelial cells and Kupffer cells more than unconstrained phospholipids.^76^

#### 3.2.4 PEG lipid modification

PEG-lipids influence several key properties, such as the size and polydispersity index of LNPs and their aggregation, and stability during both formulation and storage.^130^ Bone marrow endothelial cells transport signals to immune cells, pericytes, and hematopoietic stem cells (HSCs).^77^ For this reason, they are important target cells, but nanoparticles efficiently delivered genes to BMECs.^77^ Sago *et al.* developed efficient BMECs targeting LNPs.^77^ Changing the lipid tail of the PEG or including higher composition altered LNP's tropism and could target BMECs (Table 4).^77^

**Table 4:** Helper lipid modification

Type of lipid modified	Target organ/cells	Chemical synthesis strategy	Targeting strategy	Diseases	Reference
Cholesterol	Hepatic endothelial cells	Amine epoxide	Esterified cholesterol	X	73
Cholesterol	Liver microenvironment cells (hepatic endothelial cells and Kupffer cells)	Pyridine	Oxidized cholesterol	X	74
Phospholipid	Secondary lymphoid organs	X	Phosphatidyl serine	X	75
Phospholipid	Liver ECs and Kupffer cells	EDCI DMAP	Phospholipid containing an adamantyl group (constrained)	X	76
PEG lipid	Bone marrow endothelial cells (BMECs)	Amine epoxide	Alkyl-tailed PEG	X	77

#### 3.2.5 Various charged lipids

Many groups conducted research using nanoparticles by giving differences in charge. Small-sized and negatively charged lipid nanoparticles target lymph nodes. The size can be controlled using a microfluidic device, as described above. Cholesteryl hemisuccinate (CHEMS) which is an anionic phospholipid was mixed with other lipids to form lipid nanoparticles.^78^ DOTAP containing lipid nanoparticles led to enhanced recruitment of vitronectin, and it makes the LNPs have tropism to αvβ3 expressed cells.^79^ DODAP containing lipid nanoparticles having a negative charge target antigen-presenting cells in the spleen.^92^ For transfection of DNA to the liver, DSPC and 18PG lipids were found to be the best among DOTAP, DDAB, DOPE, DSPC, and 18PG.^93^ Replacing phospholipids with 18:1 BMP (S, R), which has a negative charge, led to spleen targeting.^91^

Siegwart's group works on a study that will explain the reasons for the use of nanoparticles by changing charges. They are the leading group researching zwitterionic, ionizable cationic, and permanently cationic helper lipids that enable organ-selective CRISPR–Cas9 gene delivery in the spleen, liver, and lungs, respectively, following intravenous administration.^94^ The group also conducted a study to figure out the mechanism. With different helper lipids, LNPs have differing p*K*_a_ and tropism, which lead them to targeting of different organs.^133^ They also developed a unique formulation platform, using other solvents as described above. The difference in viscosity between solvents makes the difference in the flow rate of the solvent. The Siegwart group focused on how to formulate selective organ targeting LNPs (Table 5).^95^

**Table 5:** Various charged lipids

Charge of the lipid	Target organ/cells	Chemical synthesis strategy	Targeting strategy	Diseases	Reference
Anionic	Spleen and liver	X	Helper lipids	X	91
Anionic	Lymph node	X	Cholesteryl hemisuccinate (CHEMS)	X	78
Permanently cationic	αvβ3-expressed cells	X	18:1 TAP (DOTAP)	Tumor (melanoma and hepatocellular carcinoma)	79
Permanently cationic	APCs in the spleen	X	DODAP	X	92
Zwitterionic, ionizable cationic, and permanently cationic	Liver	X	Mixture of lipids (DOTAP, DDAB, DOPE, DSPC, 14PA, and 18PG)	X	93
Zwitterionic, ionizable cationic, and permanently cationic (respectively)	Spleen, liver, and lungs (respectively)	X	Synthetic iPhos	X	94
Anionic, neutral, and cationic (respectively)	Spleen, liver, and lungs (respectively)	X	Anionic lipids (18PA, 14PA and 18BMP) and DOTAP	X	95

## 4. Isolation methods of cell-derived nanovesicles

Cell-derived nanovesicles (CDNs) are currently being investigated as a promising approach for cell-free therapeutics. A crucial aspect of utilizing CDNs is the efficient isolation and purification process, which should have a good yield. Furthermore, it is imperative to characterize these CDNs to assess their biological competence and ensure the reproducibility of their properties. Currently, the isolation of CDNs can be broadly categorized into two approaches. The first approach involves the isolation and purification of CDNs naturally secreted by cells. This method presents certain challenges, primarily due to the need for large-scale cell culturing over extended periods for obtaining significant yield for clinical translation studies, as each cell is known to produce approximately 50 CDNs per minute.^134^ Nevertheless, recent advancements in isolation procedures have shown promise in overcoming yield limitations. The second approach involves the application of external stimuli (such as mechanical extrusion, ultrasonication, repeated freeze–thaw cycles, and other treatments) to the host cells, which is demonstrated to enhance the production of CDNs within a shorter timeframe. Here, we attempted to provide current developments in isolation methodologies in these two approaches.

### 4.1 Isolation of naturally secreted CDNs

#### 4.1.1 Ultracentrifugation-based techniques

Researchers have explored various methodologies to isolate CDNs that are naturally secreted by cells, to achieve higher yield. Among them, the differential ultracentrifugation (DUC) technique has gained significant prominence due to its capacity to deliver superior yield, scalability, and large-scale processing capabilities. In this technique, low centrifugation forces ranging from 300 × *g* to 10 000 × *g* are applied for removing cells, cellular debris, and subcellular structures. Subsequently, higher centrifugation forces in the order of 100 000 × *g* to 200 000 × *g* are applied to isolate the CDNs.^135^ In some methodologies, the filtration technique was utilized instead of applying low centrifugation force, and furthermore, it was followed by high centrifugation force to separate CDNs.^136,137^ Recently, Tian *et al.* conducted a comparative analysis of the purity of CDNs isolated using 5 commercial kits against those isolated using the DUC technique. The analysis was performed using the lab-built nanoflow cytometry that has an analysis rate of 10 000 particles/minute with a resolution comparable to that of transmission electron microscopy.^138^ The results of the analysis revealed that CDNs isolated using DUC exhibited higher purity compared to those isolated using the commercial kits. Despite the promising findings, it is important to note that the DUC technique is time consuming (>4 hours for each round of centrifugation) and is associated with poor repeatability. Additionally, high shear force during ultracentrifugation can damage the CDNs, thereby potentially reducing the biological activity.^135^ Dash *et al.* reported that the DUC technique yielded good morphology of CDNs but the presence of aggregating particles resulted in less suspension stability and therefore produced agglomerated particles.^139^ To circumvent some of the problems, researchers utilized the density-gradient ultracentrifugation (DGUC) technique, wherein the separation medium with a gradient density (such as sucrose, glycerol and iodixanol) is used to separate particles of similar densities.^140^ Yamashita *et al.* analyzed the CDNs isolated from three different techniques: ultracentrifugation, ultracentrifugation with iodixanol as a cushion media and iodixanol based DGUC.^141^ The results showed that CDNs isolated using DGUC had narrow size distribution with higher dispersion, thus, resulting in higher recovery during sterile filtration. Additionally, the Deun *et al.* study showed that CDNs isolated using DGUC had higher purity, it terms of the highest number of positive CD63 and other exosomal marker proteins, in comparison to those isolated using ultracentrifugation and ExoQuick and total exosome isolation precipitation methods.^142^ Despite the higher purity of the isolated CDNs, this method requires preliminary preparation of density gradient solutions and much longer centrifugation times (>16 h), thereby limiting its scale-up operation for clinical translation.

#### 4.1.2 Filtration-based techniques

Ultrafiltration (UF) is one of the simple methods for isolating CDNs, which employs membrane filters of different molecular weight cut-offs to separate the undesirable cell remnants. Application of driving force for ultrafiltration can be carried out using centrifugation, pressure (mechanical force), and electric charge.^143^ For example, He *et al.* utilized low-speed centrifugation-derived UF for isolating CDNs.^144^ The authors used a 0.22 μm filter membrane with a dialysis membrane having a molecular weight cut-off of 10 MDa. This method effectively removed microvesicles and other sub-cellular remnants >200 nm. Compared to the ultracentrifugation technique, the proposed method utilized a low-speed centrifuge and it could be utilized for large-quantity samples.^144^ Pressure-derived UF could be carried out by dead-end filtration (DEF) (such as syringe filters) and tangential flow filtration (TFF). Dead-end filtration could be used only for a small sample volume, as the filter residue could rapidly block the membrane pore, resulting in lower yields.^145^ On the other-hand, TFF could emerge as a feasible solution for large volume samples, because of the perpendicular flow of the feed to the membrane. The filter residue will be carried away by the filter flow, leading to reduced clogging of membranes.^145,146^ This was observed by Dehghani *et al.* in comparing the CDN isolation procedure between TFF and DEF in ultrathin nanomembranes.^147^ Additionally, TFF samples showed a high portion of CD63-positive CDNs with minimal contamination. Similarly, Kim *et al.* also found that the TFF isolation procedure was superior to the ultracentrifugation technique based on the yield and purity.^148^ For electric charge-derived UF, Shi *et al.* developed a lab-on-a-chip device for isolating CDNs, utilizing insulator-based dielectrophoresis.^149^ Similarly, Cho *et al.* showed isolation of CDNs using an electro-migration combined TFF method, which resulted in 14 times increased recovery of CDNs compared to that by ultracentrifugation.^150^ Additional studies in simplifying the device could help in the translation of this method for large-scale isolation of CDNs.

Gel-filtration or size exclusion chromatography is a technique based on the particle diameter that helps in separation based on the molecular sizes. Briefly, the sample feed is introduced to a column packed with porous gel beads, wherein the large particles such as cell debris and remnants in the sample cannot enter the porous beads, leading to fast elution, whereas the CDNs owing to their small size are eluted slowly, thereby achieving filtration.^143,151^ Because of the simplicity, cost-effectiveness, and high purity of CDNs with biological activity, gel-filtration is one of the preferred methods. Recently, many studies have shown that the gel-filtration approach is superior in isolating CDNs with higher biological activity compared to other techniques,^152–156^ since this method utilizes gravity or a very low-speed centrifuge, which does not affect the biological activity of the CDNs. Some of the drawbacks include the dilution of CDN samples after gel-filtration, which requires additional methods such as DUC or ultrafiltration to concentrate them.^157^

#### 4.1.3 Immunoaffinity-based techniques

The membrane of CDNs is enriched with proteins and receptors, which can be used to isolate CDNs by utilizing antigen–antibody reactions. Generally, antibodies could be fixed to immunoaffinity matrices such as magnetic particles, chromatographic matrices and microfluidic devices.^158^ Depending upon the protein marker and its interaction with the antibody, an immunoaffinity matrix can be selected. For example, Mathivanan *et al.* developed isolation technique based on an epithelial cell adhesion molecule (EpCAM) and antiA33 for isolating CDNs.^159^ In addition, other prominent markers of CDNs such as CD9, CD63 and CD81 could also be utilized in isolating CDNs.^140,160^ Despite the higher purity in CDNs and a relatively quick isolation procedure, a major challenge with immunoaffinity-based techniques is recovering CDNs from the antibody-bound surfaces without denaturation. To circumvent the above-mentioned problem, Nakai *et al.* utilized a Ca^2+^-dependent Tim4 protein, which has specific binding towards phosphatidylserine that is highly expressed on the CDN surface.^161^ The Tim4 protein was immobilized onto the magnetic beads and binding with CDNs takes place in Ca^2+^-dependent media. Furthermore, the CDNs can be separated from the magnetic beads through the introduction of Ca^2+^ chelators such as EDTA. In another case, researchers utilized a chemically cleavable linker (such as 3,3′-dithiobis(sulfosuccinimidylpropionate)) between the antibody and matrix. Upon CDN binding to the antibody, CDNs can be isolated with reduction of the chemical linker with dithiothreitol which would cleave the bond, thereby releasing the CDNs for a further down-stream process.^162^ In addition, researchers also explored the use of aptamers to isolate CDNs. Aptamers are single-stranded RNA or DNA molecules that can bind to specific proteins, similar to antibodies.^163,164^ Aptamers can effectively bind to the target protein only when both of them maintain their tertiary structure, and the isolated CDNs can easily be seperated by adjusting the ionic strength and metal ion concentration to cleave the bonding between aptamers and CDNs.^165,166^ Zhang *et al.* utilized a DNA aptamer immobilized matrix for isolating CDNs having the MUC1 protein.^166^ Despite the several advantages of obtaining a highly selective class of CDNs, this method comes with a high cost, requires proper storage of reagents and cannot be utilized for large volume samples because of the cost, and certain studies do not require a specific sub-class of CDNs.

#### 4.1.4 Polymer precipitation-based techniques

Polymer precipitation utilizes polymers such as polyethylene glycol (PEG) and protamine, and protein organic solvent precipitation (PROSPR) works by reducing the dispersibility of CDNs, allowing them to precipitate with the polymer.^143,167^ This technique is well demonstrated and many commercial kits are available for the isolation of CDNs. Recently, Dash *et al.* compared the isolation techniques of CDNs, namely, PEG, PROSPR and ultracentrifugation. The results showed that the PEG-based precipitation technique is better at achieving high-quality and stable CDNs.^139^ Some of the drawbacks include co-precipitation of lipoproteins and virus particles along with CDNs, which can adversely affect the downstream analysis of the CDNs. Notably, Kırbaş *et al.* proposed a two-phase aqueous system-based precipitation technique that could circumvent some of the problems.^168^

### 4.2 Stimuli-mediated CDN preparation

Inherently the problem of slow production of CDNs by the cells could be bypassed by the application of external stimuli such as sonication, mechanical extrusion, chemical, biochemical, hypoxia, thermal, and oxidative stress to cells.^169^ This can boost the CDN production by the cells or help in the refabrication of CDNs, including disruption of host cells and their reformation into CDNs. Following these treatments, the cell membrane can undergo either bubbling-like activity, leading to the efficient release of CDNs or undergo structural and morphological changes to facilitate the generation of CDNs. Nevertheless, some of the above-mentioned methods have some disadvantages. For example, the addition of ionomycin and calcium phosphate could enhance the CDN production by ∼2.5 fold within 3 days, nonetheless concentration-dependent, and a change or increase in concentration of these molecules can cause cytotoxicity.^170^ Similarly, ionizing the cells to increase CDNs could also lead to reduced viability.^171^ Additionally, thermal and oxidative stress can lead to an increase in CDN production by 20–30 fold in 24 hours; however, they could contain immune-responsive factors in the CDNs, which can impair the therapeutic application of these CDNs.^172^ Thus, these methods can cause heterogeneity in the CDN (in proteome and lipidome) population and adverse effects in their therapeutic use, limiting their use in clinical translation.^173,174^ Hence, we are further discussing only simple exogenous applications that do not adversely affect the biological activity of CDNs.

Among the various exogenous stimuli discussed, sonication emerges as a straightforward and highly effective method for the generation of cell-derived nanovesicles (CDNs), particularly in the context of therapeutic applications. For example, Ambattu *et al.* showed a low-power, high-frequency acoustic wave stimulation (4 MHz) resulted in 1.7 to 2.1 fold per h, overall, achieving an 8–10 fold increase in CDN production for 7 cycles of acoustic wave stimulation.^175^ Studies also showed that the viability of the cells was not affected by sonication. The authors showed that the application of acoustic stimulation resulted in an elevation in intracellular Ca^2+^ levels, resulting in enhanced permeability and activation of the endosomal sorting complexes required for the transport machinery (ESCRT) pathway. Consequently, levels of CD63 and apoptosis-linked gene 2 (ALG-2) interacting protein X (ALIX) were increased, leading to increased secretion rates of CDNs. In their follow-up study, the authors showed that the application of sample high frequency, low power, and acoustic waves in mesenchymal stem cells showed an osteogenic differentiation, even with a short application of acoustic waves for 10 min daily for 5 days.^176^ Hence, the developed method can be used for therapeutic applications. Similarly, Yuana *et al.* also showed a successful increase in CDN production with the application of 1.5 MHz acoustic stimulation to cells.^177^

Taking inspiration from liposome synthesis, mechanical extrusion was utilized for increasing the production rate of CDNs from cells, which happens with the disruption of cell membranes with reorganization into CDNs. For example, Jang *et al.* utilized serial extrusion through filters with diminishing pore sizes (10, 5, and 1 μm) and displayed a 100-fold increase in CDN production, which had the characteristics similar to those of the naturally secreted CDNs.^178^ Similarly, Wan *et al.* utilized series of mechanical extrusion for preparing CDNs with metabolic engineering of cells to display an aptamer conjugated cholesterol–PEG ligand for targeted paclitaxel delivery.^179^ Furthermore, to increase CDN production, Ilahibaks *et al.* utilized both sonication and series of mechanical extrusion.^180^ Wen *et al.* carried out a detailed study for finding out the difference between exogenous stimuli-assisted CDNs and naturally secreted CDNs. The protein sequencing of membrane proteins showed ∼71% similarity between these two groups and analysis of the top 1000 small RNA showed ∼65% similarities.^181^ Thus, exogenous stimuli-assisted CDNs can be utilized for therapeutic application in similar lines with naturally secreted CDNs.^181^ Even though these methods could increase CDN production, they need to be isolated and purified through some of the techniques we described above. The increased production can help in achieving higher quantities of CDNs, overcoming some of the disadvantages of isolation techniques.

## 5. Targeting strategies of cell-derived nanovesicles

With the advent of COVID-19, LNPs have risen rapidly. Rurik *et al.* were able to target both T cells and transfect them with specific CAR-T cells by intravenous LNP injection.^85^ However, the problem of toxicity and the limitations of targeting in LNPs have been steadily raised.^11^ Cell-derived nanovesicles (CDNs) have risen sharply to replace the LNPs.^11^ In this section, how CDNs are employed as a targeted therapy will be explained.^11^

The homing of CDNs to the target site is facilitated through the inherent membrane proteins or the surface modifications of CDNs. Even though CDNs possess the native targeting ability, they may not be sufficient for targeting different cell types.^182,183^ However, it may augment the surface modifications for improved targetability and efficacy.^184^

Surface modification of CDNs is a vital strategy for targeting ability and improving their stability, retention/circulation time, and biodistribution.^185^ It can be achieved by using two basic strategies: pre-insertion/pre-modification in the parent cells and the post-insertion approach, wherein the pre-modification strategy refers to modifications made prior to disrupting CDN-generating parent cells, mainly through metabolic, genetic, and membrane-based engineering, whereas the post-insertion method involves the insertion of new ligands after the isolation of CDNs, similar to the post-insertion approach in traditional lipid nanoparticles.^185,186^

### 5.1 Pre-modification of CDNs

The pre-modification approach could be achieved by manipulating the parent cells through glycometabolic or genetic engineering (Table 6).^185^

**Table 6:** The targeting strategies: pre-modification of CDNs

The strategy of manipulation	Target organ/cells	Ligand	Disease	Reference
Glycometabolic	Blood vessels	DSPE-PEG-RGD (Arg–Gly–Asp)	X	187
Genetic	Neurons, microglia and oligodendrocytes in the brain	RVG (Rabies Virus Glycoprotein)-Lamp2b (lysosome-associated membrane protein 2b)	Alzheimer's disease	189
Genetic	Brain	RVG (Rabies Virus Glycoprotein)-Lamp2b (lysosome-associated membrane protein 2b)	Ischemic stroke	190
Genetic	Chronic myelogenous leukemia (CML) cells	Fragment of interlukin 3 (IL-3)	Acute myeloid leukemia	191
Genetic	B lymphocytes	CD 19 chimeric antigen receptor	Hematological malignant diseases	193
Genetic	Liver cancer cells	Pre S1 as a targeting moiety for sodium taurocholate cotransporting polypeptide (NTCP)	Liver cancer	194
Glycometabolic	Activated macrophages (M1)	Dextran sulfate (DS) as a targeting moiety for macrophage scavenger receptor class A (SR-A)	Rheumatoid arthritis	196
Glycometabolic	4T1	CD 47	Breast cancer	197

Genetically engineering the donor cells to specifically overexpress the target ligand in preparing targeted CDNs. In many situations, depending upon the nature of the protein, it may not be transported to the plasma membrane. To circumvent this problem, researchers transfected the cells with a plasmid that encodes a fusion protein of a plasma membrane protein with the target ligand. Thus, the fused protein will be expressed in the plasma membrane along with the target. For instance, lysosome-associated membrane protein 2b (Lamp2b) is expressed on the exosome surface and therefore, target ligands are fused to Lamp2b at the N-terminus so that they get transported to the membrane surface along with Lamp2b.^188^ For instance, rabies virus glycoprotein (RVG) is commonly used for brain targeting as it specifically binds to the nicotinic acetylcholine receptor, and plasmid encoding for RVG-Lamp2b was transfected to dendritic cells (DCs). The siRNA-loaded RVG-Lamp2b containing exosomes was able to target neurons, microglia and oligodendrocytes in the brain.^189^ Yang *et al.* also utilized RVG-Lamp2b containing CDNs for delivering circular RNA to treat ischemic stroke.^190^ The authors showed the functional benefits of developed CDNs in ischemic models in both mice and rhesus monkeys. Similarly, Bellavia *et al.* engineered HEK293T cells to express the interleukin 3 receptor (IL3R) fused to Lamp2b.^191^ The CDNs isolated from these cells were used to target chronic myelogenous leukemia (CML) cells to deliver the payload. The authors loaded imatinib or BCR-ABL siRNA in these exosomes and they were able to target the CML cells specifically and inhibit their growth, as seen from their results *in vitro* and *in vivo* studies. Instead of fusing the target ligand to the whole exosomal protein like Lamp2b, Zhang *et al.* genetically engineered HEK293T cells to display the human epidermal growth factor (hEGF) or anti-HER2 Affibody, as the targeting ligand by fusing them with hydrophobic transmembrane peptides.^192^ Thus, sorting peptides successfully presented the targeting moiety on the exterior of the plasma membrane. The authors also showed that their CDNs were having higher targeting efficacy compared to the conventional targeted liposomes, due to the ideal target ligand orientation on the membrane surface. Xu *et al.* utilized chimeric-antigen receptor (CAR) tropism for selective tumor targeting, wherein the authors established a stable HEK293T cell line that expresses CD19 CAR.^193^ The CDNs prepared from these cells were electroporated with cas9 and sgRNA for the MYC oncogene. The developed CDNs showed a higher accumulation in the Raji-bearing xenograft mice model. Red blood cells (RBCs) are one of the popular donor cells for developing CDNs as they possess lower immune recognition. However, they are anucleated cells, and thus genetic modifications are impossible. Lv *et al.* circumvented this problem by using the gene knock-in model in mice to express peptide (Asn–Gly–Arg) ligands on the surface of RBCs.^194^ The CDNs isolated from the developed transgenic mouse RBCs expressed the NGR, which was used to target the aminopeptidase N, a membrane protein present in the tumor cells.

Glycometabolic engineering is another approach for achieving the targeting of CDNs toward the cells of interest. Specifically, donor cells can be cultured in pre-treated media for a modulated expression of a specific ligand, or the lipid component ratio can be modulated or engineered to alter the structure of carbohydrates or any other component in the lipid bilayer. Through this approach, we will be able to modulate both the expression level of specific glycans and alter the chemical structure of the sugar moiety in the glycan.^195^ Specifically, this technique can introduce bio-orthogonal groups for receptor-like targeting. For instance, Wang *et al.* cultured the donor cells (K562 CML cells) in culture media containing c(RGDyK) functionalized 1,2-stearoyl-*sn*-glycerol-3-phosphoethanolamine-*N*-[RGD(polyethylene glycol)-2000] (DSPE-PEG-RGD).^187^ The donor cells would uptake the DSPE-PEG-RGD from the media and eventually, get self-assembled in the plasma membrane of the donor cells. Thus, CDNs isolated from these cells would have RGD ligands on the surface. The isolated functional CDNs were used in pro-angiogenic therapy in the zebrafish model. You *et al.* utilized metabolic glycoengineering in adipose-derived stem cells to produce exosomes that can target activated macrophages in RA and provide M1 to M2 polarization.^196^ Nie *et al.* utilized the metabolic engineering approach to produce CDNs that can avoid immune recognition and thereby increase their circulation time.^197^ Specifically, HEK293T cells were transfected with a plasmid that encodes for CD47. Biologically, CD47 would interact with signal regulatory protein-α (SIRPα) in the phagocytes and inhibit phagocytosis, thereby improving circulation time.^198,199^ In this study, M1 macrophages were allowed to grow in the azide-choline-containing media, to get azide-CDNs. Furthermore, CD47 and SIRPα were conjugated with a pH-sensitive linker containing dibenzocyclooctyne (DBCO). With click chemistry, antibodies were conjugated to the CDNs. The injected CDNs had a long circulation time, because of the “don't eat me signal” and when they get into the tumor site with an acidic microenvironment, the “don't eat me signal” gets cleaved, and allows the macrophages to exhibit effective phagocytosis, and M1 CDNs would reeducate the M2 macrophages to switch to M1, thereby achieving synergistic tumor therapy.

It should be noted that pre-modification of the CDN approach would make the parent cells direct the biosynthesis of certain target ligands and the trafficking machinery to transport these ligands to the plasma membrane without the use of any chemical cross-linking or alterations. However, this approach would be limited by controlling the density of these target ligand expressions on the plasma membrane and eventually in the CDNs.^185,200^ The topology of the target ligand needs to be carefully checked after preparation. Hence, the way forward in the pre-modification approach of CDNs would be to achieve the required target ligand density and topology in the CDNs.

### 5.2 Post-modification of CDNs

Despite non-invasive options with pre-modification of CDNs, the procedures are complex, and inhomogeneous target ligand density in CDNs and cell-level manipulation largely restrict the ligand options. In the case of the post-modification approach, the target ligands are added after the preparation/collection of CDNs. With the convenience and variety of options to choose from for inserting the target ligand in the CDNs, many strategies have been formulated (Table 7). In addition to the target ligand conjugation, post-modifications of CDNs also include modulating the rigidity of the CDNs by changing the ratio of cholesterol to other lipid membrane components, the addition of PEG molecules to elude immune recognition and to improve the dispersion stability, and so on, which can be carried out.^201–203^

**Table 7:** The targeting strategies: post-modification of CDNs

Chemical/physical strategy	Target organ/cells	Ligand	Disease	Reference
Succinimidyl-[(*N*-maleimidopropionamido)-polyethyleneglycol] ester (NHS-PEG-maleimide) is used as a hinge that anchors the ligands	PC3 cells	Recombinant human hyaluronidase, PH20 (rHuPH20)	Cancer having a pericellular HA matrix	205
DNA is engineered to be a hinge that anchors the ligands	A549 and MCF 7 cells	Quantum dots (QDs)	Lung cancer and breast cancer	206
Thiol–maleimide is used as a hinge that anchors the ligands	HepG2 cells	Antibody against a transferrin receptor (TfR)	Hepatocellular carcinoma	207
DSPE-PEG-maleimide is used as a hinge that anchors the ligands	MCF 7 cells	FITC for fluorescent imaging	Breast cancer	208
Dibenzocyclooctyne (DBCO)–azide cycloaddition	Integrin α_v_β_3_ in reactive cerebral vascular endothelial cells	c(RGDyK) peptide	Cerebral ischemia	209
Copper-catalyzed azide alkyne cycloaddition	4t1 cells	4t1 exosomal proteins	Breast cancer	210
Supplementing the membrane with additional cholesterol, stabilizing the nanostructure and facilitating the retention of a pH gradient	4t1 cells	No ligand	Breast cancer	203
Phospholipid insertion to anchor the epidermal growth factor receptor (EGFR) binding nanobodies	EGFR-overexpressing tumor cells	EGFR	X	211
Tetrahedral DNA structures that are conjugated with a DNA aptamer are tethered in CDNs using cholesterol anchoring	HepG2 cells	TLS11a aptamers	Hepatocellular carcinoma	212
ChiP in DMSO was mixed with exosomes in PBS, and the mixture was shocked in an ice bath (hydrophobic aggregation was used)		A multifunctional chimeric peptide		213
The positive charge from the multifunctional peptide could electrostatically interact with the negative charge of the CDNs	HepG2 cells	Pullulan	Hepatocellular carcinoma	214

Fundamentally, the reactive groups in proteins and ligand components of CDNs are the carboxyl group, amine group, hydrosulfonyl group, and sulfhydryl group.^200,204^ For conjugating protein molecules to CDNs, Zhou *et al.* utilized a bifunctional linker for conjugating hyaluronidase to the CDNs derived from red blood cells.^205^ Specifically, the maleimide group in the linker was conjugated to hyaluronidase through the cysteine residue and the other end of the linker contained NHS ester for amide conjugation through the membrane proteins. One of the advantages of this method is that the length of the linker can be optimized. In another study, Fan *et al.* utilized the Michael addition reaction for conjugating the biotinylated DNA hinge sequence to the CDNs derived from the M1 macrophages.^206^ Furthermore, streptavidin-conjugated quantum dots were tethered to the biotin molecule in the DNA hinge, thereby achieving DNA hinge conjugation and quantum dot tethering. Chen *et al.* utilized a mild reduction of disulfide groups in the membrane protein of CDNs isolated from milk.^207^ The mild reduction disulfide yielded free thiol groups and subsequently, the maleimide–thiol conjugation reaction was utilized to conjugate either maleimide-derivatized fluorophore or transferrin or folate receptor. In addition to the Michael-addition reaction and the amide conjugation reaction, biorthogonal reactions could also be utilized for introducing the target ligand to the CDN surface.^13,208^ Of the many biorthogonal reactions, many researchers have utilized alkyne–azide cycloaddition reactions. For instance, Tian *et al.* utilized dibenzocyclooctyne (DBCO)–azide cycloaddition to conjugate the RGD sequence to CDNs derived from the mesenchymal stem cells.^209^ Thus, the prepared RGD containing CDNs showed higher targeting properties in the ischemic region of the brain. Similarly, Smyth *et al.* conjugated 4-pentynoic acid (4PA) to the CDN surface proteins and further utilized the azide functionalized fluorophore to be conjugated to the 4PA on the CDN surface.^210^

In addition to the chemical modification approaches, physical modification techniques, such as lipid post-insertion or physical adsorption can be carried out. For example, cholesterol insertion in the CDNs improves their stability during pH variation. Zhang *et al.* post-inserted cholesterol into the CDNs derived from the RBCs at a 5% input ratio. With the cholesterol insertion in CDNs, the authors were able to remotely load the drugs using the pH variation method.^203^ Similarly, Koojimans *et al.* post-inserted phospholipids into the CDNs derived from mouse neuroblastoma cells and human platelets for EGFR-binding nanobodies.^211^ They found that 40 °C was optimal for insertion into the CDNs, striking a balance between the CDN's stability and insertion efficiency. On increasing the temperature to 60 °C, it was found that CDNs lost their stability and also caused protein denaturation. Zhaung *et al.* developed tetrahedral DNA structures that are conjugated with a DNA aptamer and tethered them in CDNs using cholesterol anchoring for cell targeting.^212^ The authors found that a ratio of 1:3 for aptamers to cholesterol was effective for higher tumor accumulation in the xenograft tumor model. The authors utilized the developed nanosystem for the delivery of CRISPR–Cas9 RNA-guided endonucleases for the downregulation of WNT10B, and thus, cell-selective gene editing was achieved. Even though many strategies were formulated in post-insertion for cholesterol and other lipid components, the efficacy of the post-insertion *in vivo* condition requires special attention. Molnar *et al.* reported a low retention efficiency for cholesteryl PEG lipid insertion *in vivo*.^201^ Yu *et al.* found that the length of the PEG chain and the mechanical stress affects the retention efficiency of the inserted amphiphiles into the CDNs.^202^ Higher stability was achieved for a shorter PEG length. Hence, researchers need to study the dynamic stability of the inserted molecules. Besides lipid insertion, the physical adsorption technique is also utilized to attach the target ligand to the CDNs. For example, Cheng *et al.* utilized a multifunctional chimeric peptide with an alkyl chain, a porphyrin complex-based photosensitizer, and a nucleus translocation peptide.^213^ The positive charge from the multifunctional peptide could electrostatically interact with the negative charge of the CDNs and the long alkyl group was able to tightly tether to the lipid bilayer. Similarly, Tamura *et al.* utilized spermidine-modified pullulan to tether into negatively charged CDNs.^214^ Pullulan adsorption on the CDNs improved their uptake in hepatocyte cells. The physical adsorption method is not explored by the researchers and at the same time, the stability of the CDNs also needs to be studied in detail.

## 6. Conclusion and future challenges

Despite the advancements in LNPs and CDNs, each system has distinct drawbacks. Opsonization is the most critical biological barrier. While synthetic nanoparticles like LNPs can be quickly opsonized with proteins, CDNs are much less vulnerable to opsonization.^167^ Early proteins that opsonized the surface of synthetic nanoparticles may induce fast clearances by the mononuclear phagocyte system (MPS), followed by destruction or mediation of endosomal–lysosomal pathways within those cells.^168^ Exposure of nanoparticles to blood *via* infusion may trigger complement-activation-related pseudoallergy (CARPA), which is an adverse immune overreaction (hypersensitivity).^169^ However, CDNs, made up of natural lipids, do not cause these side effects.^169^

Determining the formulation method is critical, as much as the targeting strategy is important. Whether permanently cationic lipids or ionizable cationic lipids are used tend to determine the LNP formulation method. When LNPs use ionizable cationic lipids, LNPs employing microfluidics and self-assembly processes outnumber LNPs employing thin film hydration and extrusion.^58–98^ All the LNPs with permanently cationic lipids and nearly all the LNPs without cationic lipids use thin film hydration.^99–103^ This implies the fact that the formulation method depends on the characteristics of the cationic lipids. LNPs should, therefore, be formulated after careful consideration. Also, CDNs are isolated in various ways including ultracentrifugation, filtration, immunoaffinity, polymer precipitation, and stimuli techniques.^134–181^ According to the references we mentioned, various combinations of the isolation method and targeting strategy of CDNs exist. Not only is it important to decide whether to use LNPs or CDNs to target specific cells, but it is also important to carefully determine what each individual strategy is. In this way, we can develop appropriate therapeutics having high efficacy.

Translating nanosystems into clinics has a barrier in determining appropriate manufacturing strategies that ensure quality and yield.^170,171^ CDNs have issues with scalability and reproducibility because they are natural lipids derived from cells.^172^ On the other hand, LNPs are synthetic lipid nanoparticles, and therefore they have advantages in large-scale production. COVID-19 mRNA vaccines are made using LNPs as a delivery system, meaning that clinical translation of LNPs has been successful.^173^ Using microfluidic chips, large-scale production of both CDNs and LNPs are plausible.^110,174^ Therefore, one of the future challenges will be the large-scale production of CDNs, concerning the mass production of mammalian cells to prepare for CDNs.

Trafficking *in vitro* and *in vivo* of nanosystems are critical future challenges. Most of the research on trafficking nanosystems employs fluorescent tags, either using lipid dyes or fluorescently labeled proteins. Even though these techniques demonstrated data on nanosystem absorption, the intracellular trafficking and processing of these molecules are not representative of nucleic acids.^175^ It is a field of research that has not been developed so far due to technical problems. Intracellular trafficking and determining endosomal pathways *in vitro*, needs high-resolution confocal laser scanning microscopy as well as fluorescent dye-labeled genes.^215^ Another important challenge is to find out what types of targeted cells exist after systemic delivery. Fluorescently labeled gene or fluorescent protein-expressing gene transfection was required, followed by flow cytometry analysis. However, this method has a limitation in analyzing one delivery system at a time.^85^ DNA barcoding can solve this problem and works efficiently. DNA barcoding can identify differences in species in their DNA.^177^ It is difficult to classify more than ten fluorescence types; on the other hand, there are 10 million species. Therefore, DNA barcoding enables, in a single experiment, the analysis of targeted cells by hundreds of nanoparticles.^63,64,74^

Nanosystems can target different cells without specific ligands, and the mechanism is not fully identified, as described above. In nanosystem research, the future requirement is to study the kinetics of each nanosystem. DNA barcoding for developing a targeting strategy, high-resolution confocal microscopy for identifying endosomal pathways, and large-scale production are three main future challenges. Once the challenges of the future are overcome, the future of nanosystems for tumors and other diseases will be bright.

## Conflicts of interest

The authors declare that they have no conflict of interests.

## Supplementary Material
